# Supplementary material for: Epidemiological and Molecular Surveillance of Aichi Virus A at Different Stages of Sewage Treatment: A One-Year Study in the Southeast of Brazil
Source: Viruses. 2025 May 21;17(5):736. doi: 10.3390/v17050736 (PMC12115472; doi:10.3390/v17050736)
Supplement: Supplementary file 1 [file viruses-17-00736-s001.zip › viruses-3576033-supplementary.pdf]

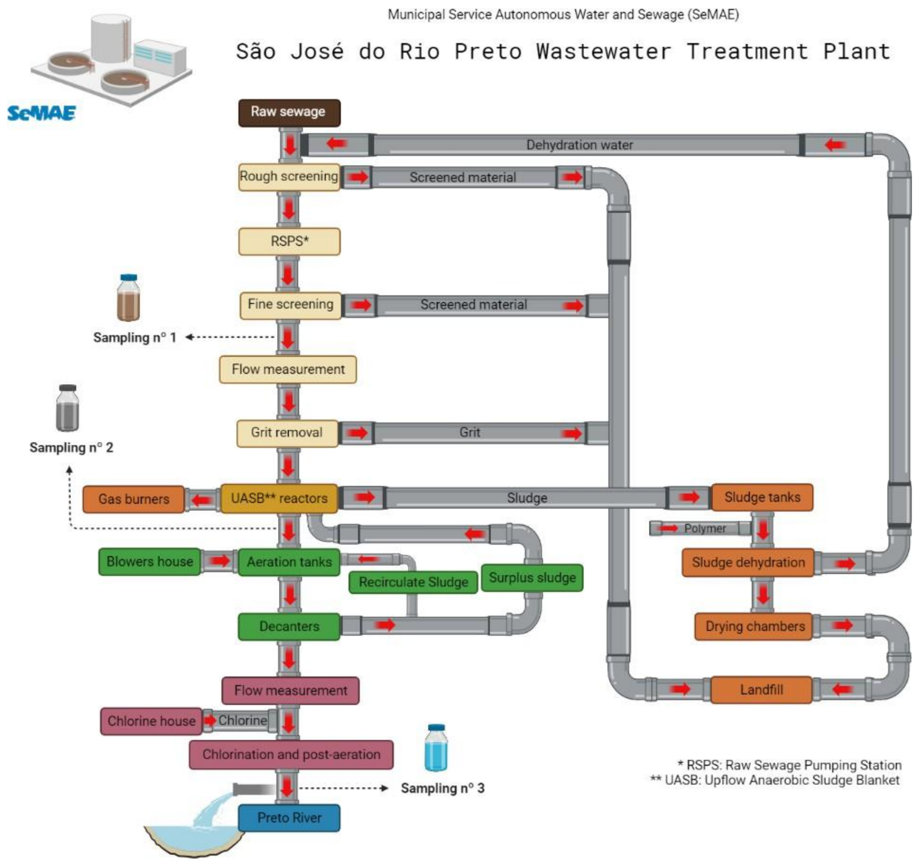

Figure S1. The flowchart illustrates the sewage treatment process in São José do Rio Preto, São Paulo state, Brazil, including the sample collection points: Sampling N1: Raw sewage sample collected after mechanical screening; Sampling N2: Sewage sample collected post-anaerobic biological treatment. Sampling N3: Sewage sample collected post-chemical treatment. ADAPTED from SeMAE, 2024 [1].

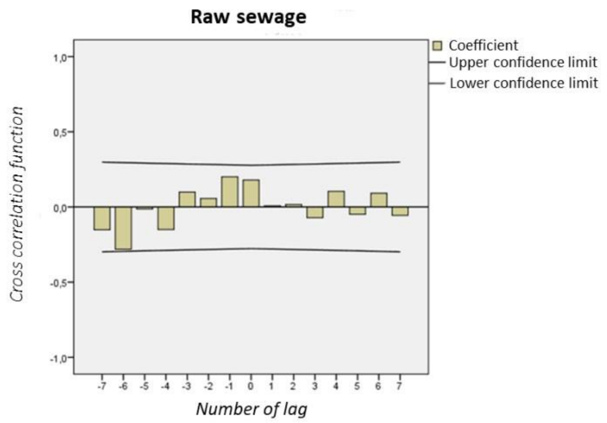

Lag	-7	-6	-5	-4	-3	-2	-1	0	1	2	3	4	5	6	7
Cross correlation function	-0.151	-0.281	-0.014	-0.150	0.099	0.056	0.201	0.179	0.008	0.16	-0.072	0.103	-0.049	0.091	-0.55
Standard error	0.149	0.147	0.146	0.144	0.143	0.141	0.140	0.139	0.140	0.141	0.143	0.144	0.146	0.147	0.149

Figure S2. Instantaneous correlation and cross-correlation between ADD cases and AiV-A viral quantification in RS, with each lag representing a week. Note: The standard error is based on the

assumption that the series are not intercorrelated and that one of the series is white noise.

Table S1. Minimum, maximum, and average temperatures, temperature variations across different months and seasons, as well as precipitation levels, reported for the city of São José do Rio Preto, São Paulo state, Brazil.

Month	Season	Minimum temperature (°C)	Maximum temperature (°C)	Average temperatures (°C)	Temperature variations (°C)	Precipitation (mm)
January	Summer	19.0	36.0	26.0	17.0	125.5
February		19.0	34.6	25.2	15.6	133.2
March	Summer/Autumn	15.2	33.9	25.6	18.7	72.4
April	Autumn	12.0	34.0	23.7	22.0	34.2
May		7.7	35.9	21.5	28.2	23.1
June	Autumn/Winter	4.9	31.6	21.4	26.7	23.0
July	Winter	4.0	32.1	20.8	28.1	4.1
August		7.0	35.0	23.0	28.0	19.4
September	Winter/Spring	15.0	39.9	27.4	24.9	18.5
October	Spring	15.0	40.9	25.9	25.9	103.4
November		14.0	37.0	26.1	23.0	84.9
December	Spring/Summer	16.0	35.0	25.4	19.0	118.8

ADAPTED from Clima Today, 2022 [2].

Table S2. The primers, probes and cycling parameters used in the present study for AiV qPCR, PCR, Nested PCR and sequencing assays, and the qPCR inhibition test (*Sketa22*).

Targets	Methods	Oligonucleotides sequences (5′- 3′)	Amplicon size (bp)	Region	Cycling	References
Aichi virus	qPCR (AiV-A)	F: GCGACCTTCGAAGGTCTGT<br>R: GTTGACGTTGACGCCAGG<br>P: FAM-CCCATCCTCGGCCAAATCACTCAA-BHQ1	210	3CD junction	10 min at 95 °C and 40 cycles of 15 s at 95 °C, 30 s at 56 °C and 30 s at 72 °C[a]	do Nascimento et al., 2024 [3]
	PCR (1st round)	F: ACACTCCCACCTCCCGCCAGTA<br>R: AGGATGGGGTGGATRGGGCAGAG	313		5 min at 94 °C and 35 cycles of 30 s at 94 °C, 30 s at 42 °C and 1 min at 72 °C, and 5 min at 72 °C	Oh et al., 2006 [4]
	Nested PCR (2nd round) Sequencing	F: GTACAAGGACAATGCGGCG<br>R: CCTTCGAAGGTCGCGGCRCGGTA	180	3C	5 min at 94 °C and 35 cycles of 30 s at 94 °C, 30 s at 38 °C and 1 min at 72 °C, and 5 min at 72 °C<br>NA	
Sketa22 (Oncorhynchus keta)	qPCR	F: GGT TTC CGC AGC TGG G<br>R: CCG AGC CGT CCT GGT CT<br>P: FAM-AGTCGCAGGCGGCCACCGT-TAMRA	77	Internal transcribed spacer region 2 (Ribosomal	10 min at 95°C, followed by 40 cycles of 15 s at 95°C and 45 s at 63°C	Haugland et al., 2005 [5]

RNA gene operon)

R: A or G; NA: not applicable [a]: The original manuscript includes the RT step, however, since cDNA was used in this study, this step was excluded from the cycling process.

Table S3. Prevalence of AiV across different seasons and stages of sewage treatment, along with the number of reported acute diarrheal disease (ADD) cases in the city of São José do Rio Preto, São Paulo state, Brazil.

Sewage samples (sampling dates)	Season	Nested PCR	AiV-A qPCR No. of positives/no. of replicates (Mean $\log_{10}$ GC/mL ± SD)	Number of reported cases of ADD in São José do Rio Preto, SP, during the sampling week[a]	Sanger sequencing Consensus sequence length (bp)	Phylogenetic analysis	GenBank access number
Raw sewage stage							
RS-1 (28/03/2022)	Autumn	+	3/3 (3.77 ± 0.07)	529	179	Y	PV101042
RS-2 (04/04/2022)		+	3/3 (4.16 ± 0.03)	523	179	Y	PV101043
RS-3 (11/04/2022)		+	3/3 (4.41 ± 0.10)	451	173	Y	PV101044
RS-4 (18/04/2022)		+	3/3 (3.37 ± 0.06)	510	179	Y	PV101045
RS-6 (02/05/2022)		+	3/3 (3.69 ± 0.01)	492	124	Y	PV101046
RS-7 (09/05/2022)		+	3/3 (3.54 ± 0.07)	593	179	Y	PV101047
RS-8 (16/05/2022)		+	3/3 (3.68 ± 0.01)	453	178	Y	PV101048
RS-9 (23/05/2022)		+	3/3 (3.28 ± 0.12)	398	179	Y	PV101049
RS-11 (06/06/2022)		+	3/3 (3.02 ± 0.02)	424	179	Y	PV101050
RS-12 (13/06/2022)		+	3/3 (3.26 ± 0.07)	422	179	Y	PV101051
RS-13 (20/06/2022)		+	3/3 (2.05 ± 0.03)	449	179	Y	PV101052
RS-14 (27/06/2022)	Winter	+	3/3 (2.20 ± 0.28)	407	175	Y	PV101053
RS-15 (04/07/2022)		+	3/3 (NQ)	460	179	Y	PV101054
RS-16 (11/07/2022)		+	3/3 (2.31 ± 0.06)	501	179	Y	PV101055
RS-17 (18/07/2022)		+	3/3 (2.22 ± 0.11)	426	179	Y	PV101056
RS-18 (25/07/2022)		+	3/3 (2.99 ± 0.13)	408	178	Y	PV101057
RS-19 (01/08/2022)		+	3/3 (2.63 ± 0.12)	457	-	N	-
RS-20 (08/08/2022)		+	3/3 (2.91 ± 0.08)	379	177	Y	PV101058
RS-21 (15/08/2022)		+	3/3 (3.20 ± 0.13)	490	177	Y	PV101059
RS-22 (22/08/2022)		+	3/3 (3.57 ± 0.05)	387	178	Y	PV101060
RS-23 (29/08/2022)		+	3/3 (3.29 ± 0.04)	488	178	Y	PV101061
RS-24 (06/09/2022)		+	3/3 (2.93 ± 0.05)	455	170	Y	PV101062
RS-25 (13/09/2022)		+	3/3 (2.33 ± 0.06)	408	179	Y	PV101063

RS-26 (20/09/2022)	Spring	+	ND	478	-	N	-
RS-27 (27/09/2022)		+	2/3 (NQ)	433	174	Y	PV101064
RS-28 (03/10/2022)		+	3/3 (3.10 ± 0.04)	451	175	Y	PV101065
RS-29 (10/10/2022)		+	3/3 (2.84 ± 0.12)	328	179	Y	PV101066
RS-30 (17/10/2022)		+	3/3 (2.25 ± 0.13)	394	-	N	-
RS-31 (24/10/2022)		+	3/3 (2.52 ± 0.23)	412	-	N	-
RS-32 (31/10/2022)		+	3/3 (2.59 ± 0.16)	385	-	N	-
RS-34 (14/11/2022)		+	3/3 (2.53 ± 0.16)	467	-	N	-
RS-35 (21/11/2022)		+	3/3 (NQ)	472	179	Y	PV101067
RS-36 (28/11/2022)		+	3/3 (2.87 ± 0.18)	434	-	N	-
RS-37 (05/12/2022)		+	2/3 (NQ)	400	179	Y	PV101068
RS-38 (12/12/2022)		+	3/3 (2.26 ± 0.20)	446	-	N	-
RS-39 (19/12/2022)		+	2/3 (NQ)	484	179	Y	PV101069
RS-41 (02/01/2023)	Summer	+	3/3 (3.38 ± 0.07)	485	-	N	-
RS-43 (16/01/2023)		+	3/3 (3.73 ± 0.04)	495	178	N	-
RS-44 (23/01/2023)		+	3/3 (3.97 ± 0.04)	486	180	Y	PV101070
RS-45 (30/01/2023)		+	3/3 (4.59 ± 0.03)	564	-	N	-
RS-46 (06/02/2023)		+	3/3 (4.42 ± 0.01)	592	179	Y	PV101071
RS-47 (13/02/2023)		+	3/3 (4.43 ± 0.05)	583	-	N	-
RS-48 (20/02/2023)		+	3/3 (4.64 ± 0.02)	846	-	N	-
RS-49 (27/02/2023)		+	3/3 (4.30 ± 0.02)	664	-	N	-
RS-50 (06/03/2023)		+	3/3 (4.23 ± 0.05)	817	-	N	-
RS-51 (13/03/2023)		+	ND	698	178	Y	PV101072
RS-52 (21/03/2023)	Autumn	+	3/3 (4.41 ± 0.06)	648	-	N	-
Post-anaerobic biological treatment stage							
PABT-2 (04/04/2022)	Autumn	+	3/3 (2.47 ± 0.15)	523	173	Y	PV101073

PABT-3 (11/04/2022)		+	3/3 (3.06 ± 0.02)	451	-	N	-
PABT-4 (18/04/2022)		+	3/3 (3.50 ± 0.05)	510	178	Y	PV101074
PABT-6 (02/05/2022)		+	3/3 (4.03 ± 0.08)	492	174	Y	PV101075
PABT-7 (09/05/2022)		+	3/3 (3.27 ± 0.14)	593	178	Y	PV101076
PABT-8 (16/05/2022)		+	3/3 (2.39 ± 0.11)	453	178	Y	PV101077
PABT-10 (30/05/2022)		+	3/3 (3.02 ± 0.11)	680	-	N	-
PABT-11 (06/06/2022)		+	3/3 (3.25 ± 0.12)	424	176	Y	PV101078
PABT-12 (13/06/2022)		+	3/3 (2.31 ± 0.11)	422	171	Y	PV101079
PABT-13 (20/06/2022)		+	2/3 (NQ)	449	178	Y	PV101080
PABT-14 (27/06/2022)		+	3/3 (3.08 ± 0.09)	407	179	Y	PV101081
PABT-15 (04/07/2022)		+	ND	460	-	N	-
PABT-16 (11/07/2022)		+	ND	501	176	Y	PV101082
PABT-17 (18/07/2022)		+	3/3 (2.57 ± 0.21)	426	179	Y	PV101083
PABT-18 (25/07/2022)		+	3/3 (NQ)	408	179	Y	PV101084
PABT-19 (01/08/2022)	Winter	+	3/3 (NQ)	457	179	Y	PV101085
PABT-20 (09/08/2022)		+	3/3 (2.92 ± 0.00)	379	179	Y	PV101086
PABT-21 (15/08/2022)		+	3/3 (3.17 ± 0.06)	490	179	Y	PV101087
PABT-22 (22/08/2022)		+	3/3 (2.87 ± 0.02)	387	179	Y	PV101088
PABT-23 (29/08/2022)		+	3/3 (2.62 ± 0.24)	488	179	Y	PV101089
PABT-24 (06/09/2022)		+	3/3 (NQ)	455	-	N	-
PABT-25 (13/09/2022)		+	2/3 (NQ)	408	178	Y	PV101090
PABT-26 (20/09/2022)		+	3/3 (2.67 ± 0.07)	478	-	N	-
PABT-27 (28/09/2022)	Spring	+	ND	433	179	Y	PV101091
PABT-30 (17/10/2022)		+	3/3 (2.92 ± 0.15)	394	179	Y	PV101092

PABT-31 (24/10/2022)		+	3/3 (2.35 ± 0.31)	412	-	N	-
PABT-38 (12/12/2022)		+	ND	446	-	N	-
PABT-39 (19/12/2022)		+	2/3 (NQ)	484	-	N	-
PABT-40 (26/12/2022)		+	2/3 (2.31 ± 0.40)	513	179	Y	PV101093
PABT-41 (02/01/2023)		+	ND	485	178	Y	PV101094
PABT-42 (09/01/2023)		+	3/3 (3.44 ± 0.03)		-	N	-
PABT-43 (16/01/2023)		+	3/3 (3.54 ± 0.06)	495	127	Y	PV101095
PABT-44 (23/01/2023)		+	3/3 (3.02 ± 0.59)	486	179	Y	PV101096
PABT-45 (31/01/2023)	Summer	+	3/3 (3.71 ± 0.03)	564	179	Y	PV101097
PABT-46 (06/02/2023)		+	3/3 (3.79 ± 0.02)	592	179	Y	PV101098
PABT-47 (14/02/2023)		+	3/3 (3.77 ± 0.04)	583	150	Y	PV101099
PABT-48 (20/02/2023)		+	3/3 (3.96 ± 0.01)	846	179	Y	PV101100
PABT-49 (27/02/2023)		+	3/3 (4.72 ± 0.03)	664	-	N	-
PABT-50 (06/03/2023)		+	3/3 (4.41 ± 0.10)	817	176	Y	PV101101
PABT-51 (13/03/2023)		+	3/3 (3.50 ± 0.16)	698	179	Y	PV101102
PABT-52 (21/03/2023)	Autumn	+	3/3 (4.26 ± 0.09)	648	179	Y	PV101103
Post-chemical treatment stage							
PCT-1 (28/03/2022)		+	ND	529	-	N	-
PCT-3 (11/04/2022)		+	ND	451	-	N	-
PCT-8 (16/05/2022)		+	ND	453	-	N	-
PCT-9 (23/05/2022)		+	ND	398	178	Y	PV101104
PCT-10 (30/05/2022)	Autumn	+	3/3 (2.48 ± 0.07)	680	179	Y	PV101105
PCT-11 (06/06/2022)		+	2/3 (NQ)	424	178	Y	PV101106
PCT-12 (13/06/2022)		+	ND	422	-	N	-
PCT-13 (20/06/2022)		+	ND	449	178	Y	PV101107
PCT-14 (27/06/2022)	Winter	+	ND	407	179	Y	PV101108

PCT-15 (04/07/2022)		+	ND	460	179	Y	PV101109
PCT-16 (11/07/2022)		+	ND	501	173	Y	PV101110
PCT-17 (18/07/2022)		+	ND	426	179	Y	PV101111
PCT-18 (25/07/2022)		+	ND	408	174	Y	PV101112
PCT-19 (01/08/2022)		+	ND	457	179	Y	PV101113
PCT-20 (09/08/2022)		+	ND	379	-	N	-
PCT-21 (15/08/2022)		+	ND	490	-	N	-
PCT-22 (22/08/2022)		+	ND	387	179	Y	PV101114
PCT-23 (29/08/2022)		+	ND	488	-	N	-
PCT-24 (06/09/2022)		+	ND	455	179	Y	PV101115
PCT-25 (13/09/2022)		+	ND	408	179	Y	PV101116
PCT-26 (20/09/2022)		+	2/3 (NQ)	478	-	N	-
PCT-28 (03/10/2022)		+	ND	451	179	Y	PV101117
PCT-29 (10/10/2022)		+	ND	328	179	Y	PV101118
PCT-32 (31/10/2022)	Spring	+	ND	385	178	Y	PV101119
PCT-33 (07/11/2022)		+	ND	406	174	Y	PV101120
PCT-34 (14/11/2022)		+	ND	467	179	Y	PV101121
PCT-40 (26/12/2022)		+	ND	513	-	N	-
PCT-43 (16/01/2023)		+	ND	495	-	N	-
PCT-44 (23/01/2023)		+	ND	486	179	Y	PV101122
PCT-45 (31/01/2023)		+	ND	564	179	Y	PV101123
PCT-46 (06/02/2023)	Summer	+	2/3 (NQ)	592	179	Y	PV101124
PCT-47 (13/02/2023)		+	3/3 (2.13 ± 0.01)	583	179	Y	PV101125
PCT-48 (20/02/2023)		+	3/3 (2.85 ± 0.03)	846	-	N	-
PCT-49 (27/02/2023)		+	3/3 (2.74 ± 0.11)	664	178	Y	PV101126
PCT-50 (06/03/2023)		+	3/3 (2.41 ± 0.09)	817	178	Y	PV101127

PCT-51 (13/03/2023)		+	2/3 (2.41 ± 0.36)	698	178	Y	PV101128
PCT-52 (21/03/2023)	Autumn	+	3/3 (2.44 ± 0.12)	648	179	Y	PV101129

SD: Standard deviation; ND: Not detected; NQ: Not quantifiable; Y: Yes; N: No; +: Positive sample; -: Analysis not performed; [a]: Reported disease cases are not dependent on the treatment phase and are consistent across different stages, and are presented for visualization purposes. Only the 'raw sewage' stage was statistically correlated with the cases, as it reflects the actual circulation of viruses in the wastewater.

Table S4. Descriptive analyses of AiV-A quantification across different seasons and stages of sewage treatment.

							Percentiles		
	**Season**	***N***	**Minimum**	**Maximum**	**Mean**	**SD**	**25th**	**50th**	**75th**
Raw sewage									
AiV-A (log_{10} GC/mL)	Autumn	14	0.00	4.41	3.05	1.42	2.77	3.46	3.86
	Winter	13	0.00	3.57	2.43	0.98	2.21	2.63	3.09
	Spring	13	0.00	3.10	1.92	0.98	1.00	2.26	2.71
	Summer	12	0.00	4.64	3.14	1.93	0.84	4.10	4.43
Post-anaerobic biological treatment									
AiV-A (log_{10} GC/mL)	Autumn	14	0.00	4.26	2.32	1.49	0.75	2.75	3.33
	Winter	13	0.00	3.17	1.84	1.19	1.00	2.57	2.89
	Spring	13	0.00	2.92	0.48	1.00	0.00	0.00	0.00
	Summer	12	0.00	4.72	3.35	1.22	3.12	3.62	3.92
Post-chemical treatment									
AiV-A (log_{10} GC/mL)	Autumn	14	0.00	2.48	0.42	0.90	0.00	0.00	0.25
	Winter	13	0.00	1.00	0.08	0.28	0.00	0.00	0.00
	Spring	13	0.00	0.00	0.00	0.00	0.00	0.00	0.00
	Summer	12	0.00	2.84	1.13	1.26	0.00	0.50	2.41

50th percentile: median.

Table S5. Mann-Whitney test assessment of the relationship between rainfall occurrence and AiV-A concentrations at different stages of sewage treatment.

**Raw sewage**	
	AiV-A
U de Mann–-Whitney	289.00
Wilcoxon W	785.00
Z	-0.682
Sig. (bilateral)	0.495
Post-anaerobic biological treatment	
U de Mann–Whitney	310.50
Wilcoxon W	806.50
Z	-0.284
Sig. (bilateral)	0.776
Post-chemical treatment	
U de Mann–Whitney	324.50
Wilcoxon W	555.50
Z	-0.027
Sig. (bilateral)	0.978

References

1. SEMAE Rio Preto., 2024. Tratamento de esgoto. Available online: https://semae.riopreto.sp.gov.br/tratamento-de-esgoto.aspx (accessed on 04 January 2025).
2. Clima Today. São José do Rio Preto, São Paulo data. Available online: https://clima.today/BR/SP/Sao-Jose-do-Rio-Preto/ (accessed on 09 September 2024).
3. do Nascimento, M.C.A., Smith, W.J.M., Liu, Y., Simpson, S.L., Bivins, A., Rahal, P., Ahmed, W. Development and comparative assessment of RT-qPCR and duplex RT-LAMP assays for the monitoring of Aichi virus A (AiV-A) in untreated wastewater samples. *Sci Total Environ*. **2024,** *952*, 175440.
4. Oh, D.Y., Silva, P.A., Hauroeder, B., Diedrich, S., Cardoso, D.D., Schreier, E. Molecular characterization of the first Aichi viruses isolated in Europe and in South America. *Arch Virol.* **2006**, *151(6)*, 1199-206.
5. Haugland, R.A., Siefring, S.C., Wymer, L.J., Brenner, K.P., Dufour, A.P. Comparison of Enterococcus measurements in freshwater at two recreational beaches by quantitative polymerase chain reaction and membrane filter culture analysis. *Water. Res.* **2005**, *39*, 559–568.
